# Challenges and Prospects of Plant-Protein-Based 3D Printing

**DOI:** 10.3390/foods12244490

**Published:** 2023-12-15

**Authors:** Shivani Mittal, Md. Hafizur Rahman Bhuiyan, Michael O. Ngadi

**Affiliations:** Department of Bioresource Engineering, McGill University, 21111 Lakeshore Road, Sainte Anne de Bellevue, QC H9X 3V9, Canada; shivani.mittal@mail.mcgill.ca (S.M.); md.bhuiyan@mail.mcgill.ca (M.H.R.B.)

**Keywords:** 3D food printing, plant protein, printer parameters, texture

## Abstract

Three-dimensional (3D) printing is a rapidly developing additive manufacturing technique consisting of the deposition of materials layer-by-layer to produce physical 3D structures. The technique offers unique opportunities to design and produce new products that cater to consumer experience and nutritional requirements. In the past two decades, a wide range of materials, especially plant-protein-based materials, have been documented for the development of personalized food owing to their nutritional and environmental benefits. Despite these benefits, 3D printing with plant-protein-based materials present significant challenges because there is a lack of a comprehensive study that takes into account the most relevant aspects of the processes involved in producing plant-protein-based printable items. This review takes into account the multi-dimensional aspects of processes that lead to the formulation of successful printable products which includes an understanding of rheological characteristics of plant proteins and 3D-printing parameters, as well as elucidating the appropriate concentration and structural hierarchy that are required to maintain stability of the substrate after printing. This review also highlighted the significant and most recent research on 3D food printing with a wide range of plant proteins. This review also suggests a future research direction of 3D printing with plant proteins.

## 1. Introduction

Three-dimensional (3D) printing is a process that lays down the physical objects from a digital blueprint layer-by-layer and fuses them together [1]. Three-dimensional printing first came to light in the 1980s after which it started offering new opportunities in the fields of medicine, education, and aerospace [2]. Three-dimensional food printing is used to give customized shapes, colors, textures, and different nutritional compositions to the food products. In particular, it can be used to design food for target populations that require personalized meals.

In 3D printing, the ink is a crucial component. A number of inks have been formulated to give different customized shapes to printed food products which can be made of complex formulations such as fruits, vegetables, animal products, and dairy. Amongst a broad spectrum of materials, plant protein is gaining attention as a raw material used in 3D printing to produce meat analogs, satisfy the personalized needs of consumers, and reduce the environmental impact of livestock rearing [3]. Proteins are macromolecules comprising amino acids linked by peptide bonds (C-N) and are generally classified into fibrous (keratin, silk) and globular proteins (soy, albumin) [4]. The demand for reliable and environmentally friendly protein sources is driven by the increase in the world population. The growing awareness of the inefficiency in protein conversion during the production of meat from livestock sparked the creation of plant-based foods as an alternate source of protein. Food consumption accounts for 30% of EU (European Union) greenhouse gas emissions (GHG), and plant-based meals typically emit fewer GHGs than animal-based foods. Plant-based meat analog production is a way of mimicking meat in terms of nutrition, texture, and sensory properties [5]. Plant-based meat is a high source of protein and thus can meet high protein requirements [6]. But, in order to work with plant-based proteins as a raw material, it is important to understand the relation between raw material and the printed material to be produced.

The processes of 3D printing are as follows: designing custom shapes using computer-aided design (CAD), pre-treating the inks to have suitable rheological parameters, feeding ink capsules, slicing designs, extruding ink from the nozzle, and depositing the structure on the printed bed [7]. In accordance with the American Society of Testing and Materials (ASTM), 3D printing depends on seven technologies including selective laser sintering, direct energy deposition, material extrusion, ink jetting, sheet lamination, binder jetting, and vat polymerization; however, not all the techniques apply to plant-protein inks [4]. Food incorporation in 3D printing is a bit challenging due to the variation in physio-chemical properties [8]. Therefore, several studies classified the 3D technologies into four major categories, (1) selective laser sintering/hot-air sintering, (2) hot-melt extrusion (used to create customized 3D chocolate products, cheese, and humus) and room temperature extrusion (used for pizza printing), (3) binder jetting (used for sugar printing), and (4) inkjet printing (used for decoration or surface fill in cake, pastry, or cookie fabrication) [9].

The rheological characteristics of protein-based inks, additives, and printing conditions have affected printing results in different ways by providing printing stability, structural support, and nutrition and have been the main research topic over the years. The objective of this review was to gather and examine information on the technical specifications for 3D printing, 3D printing parameters, printing materials, and the role of proteins in 3D printing. Additionally, the current status and prospectus of different types of plant-protein-based inks were also discussed.

## 2. Trends of Plant-Protein-Based 3D Printing

**Figure 1:**
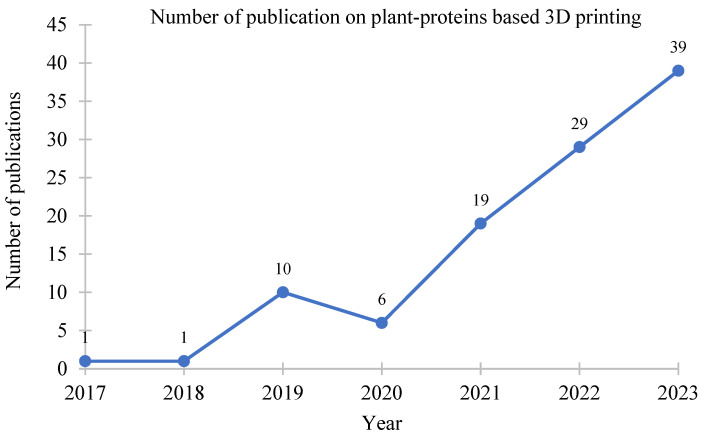
Number of scientific publications on plant-protein-based 3D printing (source: Web of Science).

Three-dimensional printing is a cutting-edge technology to design and personalize food products to cater to consumer needs and to meet market demand. Amongst the wide availability of printers, extrusion-based ones are the most commonly used ones for plant-based proteins. Plant-based foods are gaining popularity as their positive effects on human health gain wider recognition. Researchers have been exploring various plant-derived materials for 3D printing, including proteins from sources like soy, peas, and other legumes. Advances in material science contribute to the development of printable and functional plant-based materials. The number of original studies on plant-based printable materials surged to a rise in the past few years (Figure 1). This is because the current trend in 3D food printing involves providing a broader range of personalized and visually appealing food designs, utilizing digitized nutritional information to cater to specific health-focused lifestyle preferences.

Three-dimensional printing goods and services are projected to have a yearly growth rate of roughly 26% and a projected value of 40 billion US dollars by 2024 on a global scale.

## 3. Three-Dimensional Printer Parameters

**Figure 2:**
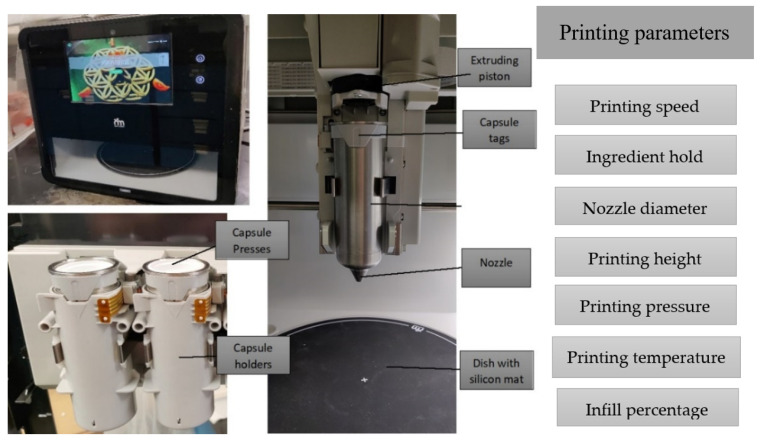
Commercial (Foodini) 3D food printer and major printing parameters.

Three-dimensional printer is the heart of the modern food industry producing personalized meals (Figure 2). Printability is one of the most important parameters in extrusion-based 3D printing and is characterized to handle dimensional stability, i.e., whether the material is capable of supporting its own weight [10]. The printability of a material (ink) is highly dependent on the properties of the food system and the 3D printer parameters used. Three-dimensional printing is not only affected by the properties, physicochemical and rheological, of the printing materials but also by the processing parameters such as the nozzle height, nozzle diameter, infill percentage, printing speed, extrusion rate, and temperature [11]. The temperature of the nozzle can affect the flowability of the material; an increase in the temperature can decrease the viscosity [12]. Past studies explored the relationship between printing parameters and the quality of 3D-printed food.

Liu et al. [13] studied the effect of the extrusion rate and printing speed on the printability of whey protein isolate (WPI) as shown in Table 1. Printing speed and extruding rate impact 3D printing simultaneously during the printing process because they alter the quantity of printed paste per unit length per unit time. It was reported that the extruding rate must be increased with increasing printing speed to feed the paste in time. Also, the printing quality decreased with the increasing printing speed.

The force applied by the commercial 3D printer (Foodini) can be modified to “hold back” the ingredient in the capsule as it moves to the first print area once the ingredient detection over the test cup is finished. The suggested default value of the ingredient hold is 4.2. It is recommended to increase the initial ingredient hold if there is an ingredient dropping from the test cup to the first print [14].

**Figure 3:**
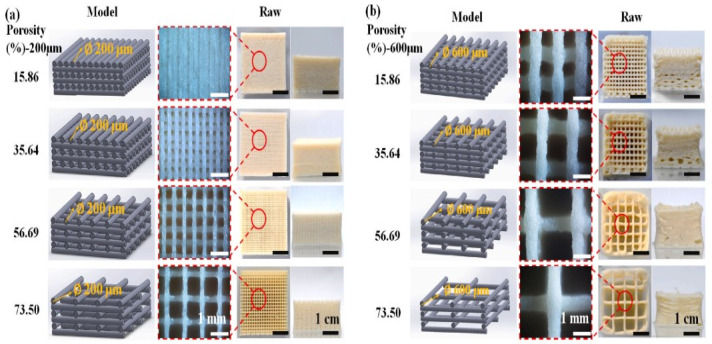
Comparison of SPI-XG-RS-based samples having different nozzle sizes and printing porosity [16].

Huang et al. [15] studied the effect of the nozzle diameter and reported that a bigger nozzle size resulted in a bigger deviation in the diameter of printed objects. Thus, decreasing the nozzle diameter would print samples closer to the designed ones. Shi et al. [16] evaluated the influence of structural geometry (nozzle diameter and porosities) of soy protein isolate–xanthan gum–rice starch (SPI-XG-RS)-based printed samples on a texture profile analysis. It was reported that the printed samples with 200 μm filaments have a higher shape fidelity than that of samples with 600 μm filaments (Figure 3). Moreover, decreasing nozzle diameter not only marks precision but also increases printing time and feed pressure. A 3D-printing system that is overloaded due to excessive printing pressure may experience machine wear. The printing procedure requires more pressure to print edible ink at lower nozzle diameters, which could lead to irregular deposition of the printable substance [17].

The nozzle height is the distance between the bottom of the nozzle and the printer bed in the printing process. The nozzle height has been identified by numerous prior studies as a significant factor influencing the printing accuracy [18]. However, Yang et al. [19] have carried out a number of thorough experiments to confirm that the nozzle height should be the same as the nozzle diameter in the 3D-printing process.

**Figure 4:**
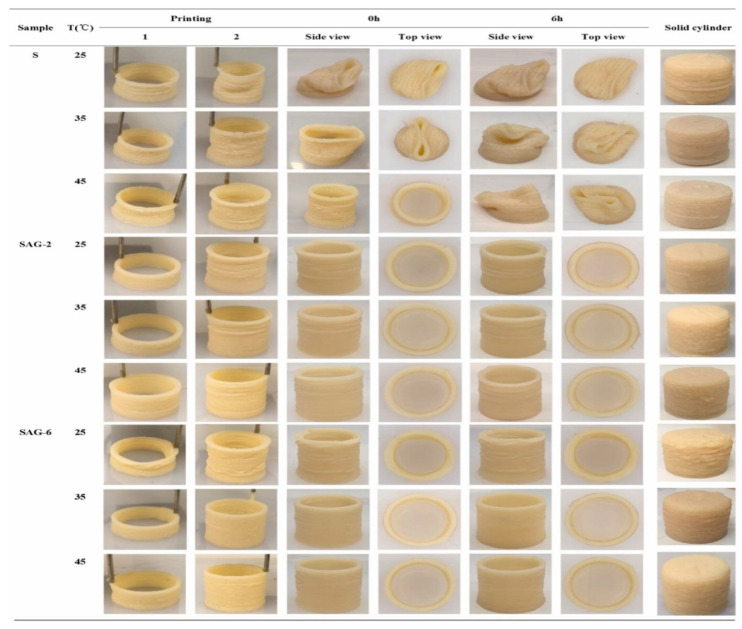
The 3D printing behavior of SPI-based pastes at 25, 35, and 45 °C (12). (S: control; SAG-2: 2% gelatin, 0.5% sodium alginate; SAG-6: 6% gelatin, 0.5% sodium alginate).

Printing temperature in 3D food printing is an important aspect influencing the rheological characteristics of food, which is likely to have an impact on the material’s 3D printability [20]. Chen et al. [12] studied the effect of three printing temperatures of 25, 35, and 45 °C on the rheological properties of SPI-based pastes. The effect of the printing temperature on the microstructure and texture of 3D-printed protein paste cylinders varied greatly according to the gelatin content in the SPI-based paste. It is reported that increasing the temperature reduced the viscosities of pastes, thus improving the rheological properties and printability (Figure 4).

**Figure 5:**
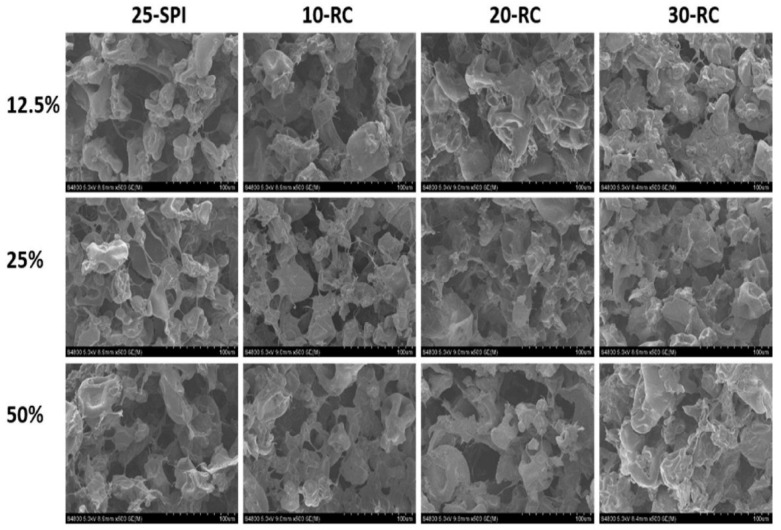
Cross-sectional SEM images for 25-SPI doughs with different RC contents as a function of infill rates (12.5, 25, and 50%).

Figure 5 shows the effect of different infill percentages (12.5, 25, and 50%) on the inner structure and post-stability of the soy protein isolate (SPI)-red cabbage (RC) inks. It was reported that the interior structure of the samples was unaffected by the various infill percentages (12.5, 25, and 50%). As for the dough composition, the increase in RC concentration reduced the number of cavities and made the structure more compact [21].

## 4. Technological Feasibility of Protein-Based 3D Printed Food

The 3D printing of plant protein presents an opportunity to expand additive manufacturing applications in the food industry. High precision characteristics of 3D printing give a way to produce plant-based meat which is subjected to mimic the taste, texture, appearance, and nutritional values of traditional meat. Amongst these, the texture still remains the challenging one [22]. So, for this, technological feasibility plays a major role. In terms of printer-related challenges, the main technological considerations for 3D printing are the dispensing mechanism and the 3D positioning method. The designing software (CAD) controls the positioning system that creates 3D structures. In the case of the dispensing system, the extruder type, which can have a single or a double nozzle, is the most common [23]. Furthermore, different operational settings may be required depending on the type of material. Three-dimensional printing of food products is limited due to the lack of suitable materials for printing because of the instability of plant-based proteins. These challenges can be overcome by taking care of the technical requirements of 3D printing.

**Figure 6:**
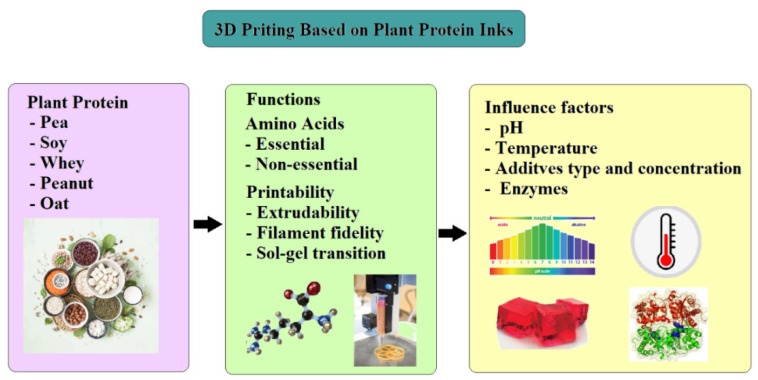
Steps to be considered while 3D food printing: sources of plant protein, functions, and influencing factors.

In addition to processing parameters and sources of protein, the rheological property of printing ink plays a pivotal role in deciding the successful printing according to the present pattern and is related to the accuracy and results of the printing (Figure 6). Viscosity plays a major role in rheology in the self-supporting and stacking properties of materials while printing [24]. Three-dimensional printing involves the extrusion of material from the nozzle in order to deposit on the surface. The ink is required to present a shear-thinning behavior, i.e., less viscosity during extrusion so that it can be easily extruded from the nozzle; however, it is expected to regain its viscosity and maintain the structure after deposition [25,26]. The viscoelastic properties of the ink, measured by a series of rheological tests, have a significant role in determining the printing performance, including the extrudability, filament fidelity, and sol-gel transition [4]. Xu et al. [27] studied the effect of enzyme-assisted apricot polysaccharide (EAP) on soybean protein isolate (SPI) gel preparation. It was reported that the dynamic rheological properties, i.e., the viscoelasticity of gels, are related to the printing accuracy and is concentration-dependent. It was demonstrated that the degree of crosslinking of SPI-apricot polysaccharide increased with increasing EAP content, thus exhibiting stronger solid-like behavior.

### 4.1. Extrudability

**Figure 7:**
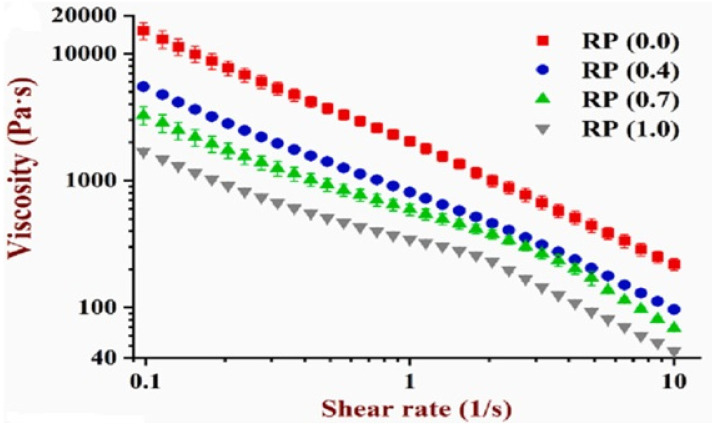
Viscosity of soy protein isolate–wheat gluten pastes with different concentrations of RP (rice protein) [29].

The efficiency with which an ink is extruded from the dispensing nozzle is termed extrudability, and viscosity is a key indicator of extrudability. Viscosity depends on the concentration of the protein isolate, molecular weight, and inter and intra-molecular interactions which are influenced by factors such as temperature, protein concentration, ion strength, and pH. Viscosity is inversely proportional to shear rate, shown by the rheological flow curve called shear-thinning behavior, which is necessary for 3D printing. For example, Yu et al. [28] reported that the viscosity of the inks decreased with the addition of polysaccharides such as guar gum and xanthan gum into the soy protein isolate (SPI) emulsion gels, thus exhibiting shear-thinning behavior. Another study used SPI-WG-RP (soy-protein isolate-wheat gluten-rice protein) pastes and reported a decrease in apparent viscosity with an increasing rice-protein ratio [29]. Also, in accordance with the same study, it could be seen that the apparent viscosity decreased with the increasing shear rate for all types of ink as shown in Figure 7 [29]. Also, various pre- or post-treatments can improve the viscosity of the sample. For instance, a study reported the effect of microwave pre-treatment on 3D printing of soy–strawberry ink resulted in an increase in the viscosity, which is more suitable for 3D food printing [30].

The structure of the material largely depends on the pH of the solution. Protein denaturation, protein–protein, and protein–water interactions are affected by pH and proper pH can prevent the collapse of the gel network from charge repulsion. It is observed that a stable printing system requires pH away from the isoelectric point of the protein towards the alkaline region (pH −7 to 10). The protein molecules aggregate at the isoelectric point (pI) in the presence of both charges resulting in the decreased efficiency. This affects the gelation property of the ink. However, similar charges increase the efficiency of the inks by repelling each other [31].

### 4.2. Filament Fidelity

**Figure 8:**
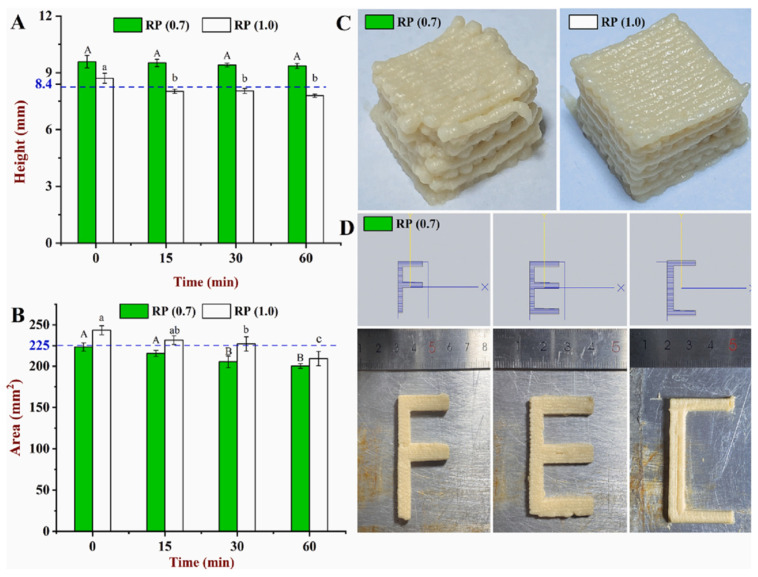
Evaluation of printing fidelity. (**A**) Height as a function of time. (**B**) Surface area as a function of time. (**C**) The image of printed cuboid using RP (0.7) and RP (1.0). (**D**) The image of three printed “English Alphabets” (25 mm × 25 mm × 4.2 mm) using RP (0.7) [29]. Upper-case and Lower-case letters represent significant difference between RP (0.7) and RP (1.0) samples, respectively.

Filament fidelity is the maintenance of the structure of the extruded material to prevent collapse and sagging. It is related to at least two viscoelastic properties, i.e., yield stress and thixotropy [4]. Insufficient yield stress leads to the collapse of the extruded material under its own weight, so the bulking agents and the thickeners such as food hydrocolloids are added for the stability of the structure. Qiu et al. [29] used different concentrations of rice protein in the SPI-WG ink to check the printing performance and reported that inks having (RP 0.7 and RP 1.0) could be successfully printed into layers. The study also reported RP (0.7) ink with the best print fidelity (Figure 8A–D). Also, Chen et al. [12] studied the printing properties of ink formulations containing textured soy protein (TSP) and drawing soy protein (DSP) with different hydrocolloids and reported that TSP with xanthan gum showed the best printing characteristics and maintained the structure during the printing of steak-like foods. Also, a high protein content increases the yield stress efficiency of the printing matrix [31]. Another study by Lille et al. [1] found that the good shape stability of an oat and faba protein isolate was achieved by high yield stress.

**Figure 9:**
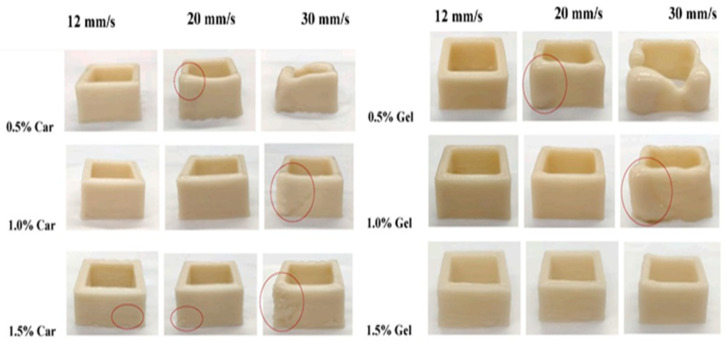
Evaluation of print fidelity of peanut protein-based inks as a function of concentration and printing speed [32].

Lin et al. [32] reported the effect of the concentration of additives and the printing speed on the fidelity of printed peanut protein. The study showed that a small amount of carrageenan (0.5%) can print objects with high fidelity at the slowest printing speed (12 mm/s speed). It was also reported that the fidelity of the printed product decreases with the increasing printing speed. Similar patterns were seen in the fidelity of the items printed with 0.5% gellan gum at various printing rates (Figure 9).

**Figure 10:**
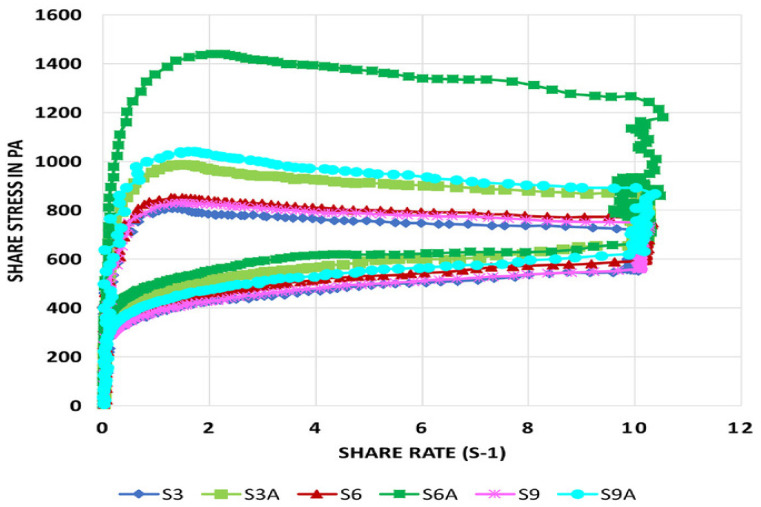
Evaluation of thixotropy of SPAH-agar inks for 3D printing. Note: S3 (3 g soy), S6 (6 g soy), S9 (9 g soy), S3A (3 g soy and 0.2 g agar), S6A (6 g soy and 0.2 g agar), and S9A (9 g soy and 0.2 g agar) [33].

Another property is thixotropy, which is the time-dependent process of rebuilding a molecular structure. It tells us whether the viscosity recovered. High thixotropy requires the highest energy to break down the internal structure, with a high resistance to time-dependent flow and high levels of internal viscosity and stability [33]. Mirazimi et al. [33] studied varying shear rates to characterize the effects of soy protein acid hydrolysate (SPAH) and agar and reported that formulation with 6 g SPAH and 0.2 g agar (S6A) exhibited the highest degree of thixotropy (Figure 10). According to Clark et al. [34], the addition of collagen and gelatin recovered 75% of the storage modulus within one second whereas, ink with alginate and methylcellulose (MC) showed 56% recovered viscosity after 30 s [35].

### 4.3. Sol–Gel Transition

Protein molecule crosslinking is frequently linked to the sol–gel transition in 3D printing, which occurs when liquid phases transform into solid phases. This crosslinking is defined by the ratio of storage moduli (G′) to loss moduli (G″), where G′ and G″ describes the elastic (solid-like) and viscous (liquid-like) properties of the ink, respectively. The sol–gel transition takes place when G′ > G″ [4]. The sol–gel transition is evaluated using a frequency sweep to give insights into the self-supporting behavior of protein inks after deposition.

**Figure 11:**
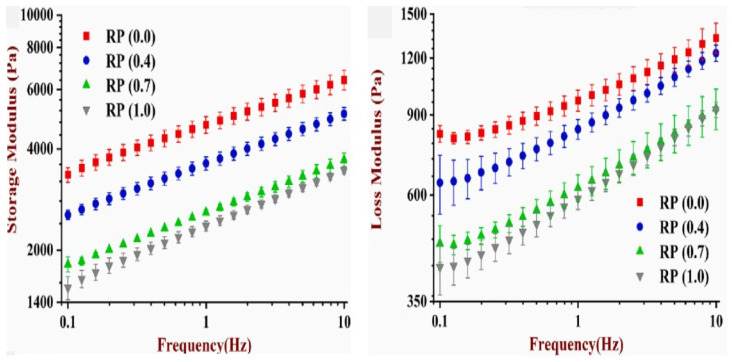
G′ and G″ of SPI-WG pastes with different concentrations of RP (rice protein) [29].

The storage modulus is used to measure the solid elastic behavior of the sample, which reflects the mechanical strength of the sample, whereas the loss modulus reflects the liquid behavior of the samples. G′ and G″ depend on the frequency. A study reported the effect of the frequency on the storage and loss moduli of SPI-WG-RP-based ink. It was concluded that both G′ and G″ values gradually increased with increasing oscillatory frequency, which is consistent with an increase in the internal friction at higher frequencies. It was also seen that G′ > G″ indicating that the soy protein-based ink exhibited predominantly elastic properties (Figure 11) [29].

The sol–gel transition is also related to the protein cross-linking which is influenced by the addition of enzymes and heating treatment. Transglutaminase is the widely used enzyme that causes protein-gel formation [36]. For example, L-cysteine hydrochloride breaks the disulfide bonds of protein, thus exposing sites for the action of transglutaminase (T_Gase_). This leads to the formation of polymers and increased viscosity for optimizing printing ability [28]. Also, adding an alginate solution of 80% to 20% pea protein solution can increase the mechanical strength and consistency of printing [37]. Furthermore, the gel strength and elasticity of the dough can be improved by the addition of fat as it promotes the uniform distribution of fat and gluten protein to obtain a more stable network [19].

The rheological characteristics of inks are highly influenced by the heating time. Yu et al. [28] reported the G′ value increases with an increase in preheating time thus exhibiting sol–gel transition. It is also influenced by the temperature. The temperature has a huge effect on the final printing effect. High-temperature protein denaturation exposes hydrophobic sites for covalent bonding [38]. A study found that the viscosity of SPIs increases with increasing the heating time to 20 min, 25 min, and 30 min, thus increasing the sol–gel transition rate [28]. Also, the 3D printability of protein pastes with different formulations can be improved by adjusting the printing temperature. The printing temperature has a significant impact on the microstructure and texture of printed food. A study revealed that with the increasing printing temperature, the hardness and chewiness of the objects made of S (soy-based), SAG-2 (soy-gelatin-sodium alginate based with 2 g gelatin), and SAG-6 (6 g gelatin) increased significantly [12].

## 5. Plant-Based Proteins for Extrusion-Based 3D Printing

**Figure 12:**
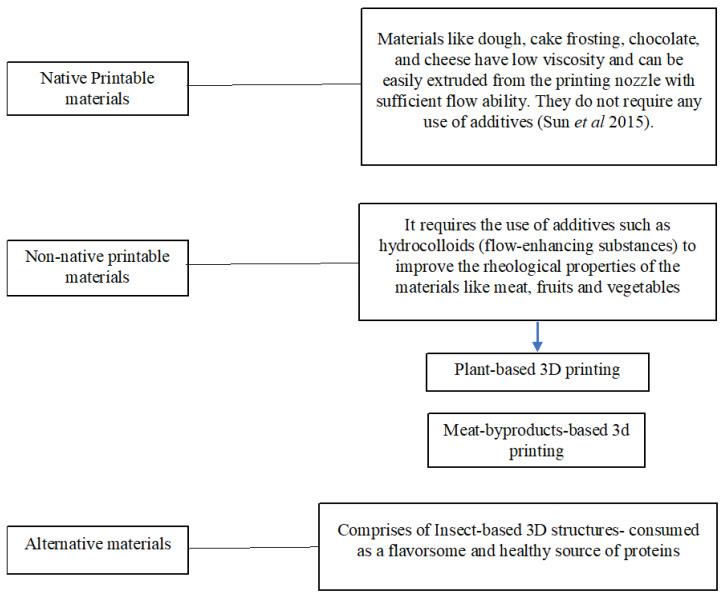
Material-based 3D food printing [22].

Extrusion-based 3D printing has been most commonly adapted in the food sector. It involves the extrusion of liquid or semi-solid material from the printing nozzle, moving in the x, y, and z-direction. One benefit of adopting extrusion-based printing is that it is able to print a wide range of materials at the same time to produce a whole meal [39]. Materials in 3D printing are broadly classified into three categories [22]—native printable materials, non-native printable materials, and alternative materials, such as insect-derived 3D structures (Figure 12). However, the increasing demand of plant-based proteins as a substitute for animal-based proteins has been a topic of research for a while now due to increasing awareness of the health benefits associated with plant proteins and of environmental concerns, i.e., reducing the environmental footprint, waste, and demand for water and energy [40]. Plant-based proteins are explored commercially to extract isolates because of their unique nutritional (metabolism and growth) and health-promoting attributes such as functionality, sensory characteristics, and labeling. Zhang et al. [41] reported soy as the most common raw material for many plant-based foods owing to its nutritional benefits; however, more recently, pea was introduced as an alternative protein that is gluten-free and due to its low allergenicity [42]. However, compared to soy, peas can be grown in more moderate climates [42]. Pea protein is a good source of fiber, starch, vitamins, minerals, and phytochemicals. However, its gelling capacity is lower than soy protein, thereby requiring the use of various additives such as hydrocolloids, carbohydrates, and lipid additives. Additives have a long history of application in food, which have the capability of alternating the properties of various natural food gels which alone have poor printing performance which is discussed later in the section.

### 5.1. Role of Plant Protein

There has been considerable research into the use of plant proteins for the formation of 3D printable inks, especially meat analogues (Table 3). The formation of protein-based feed focuses on material formation methods in accordance with the final product printed. For instance, the printing of meat analogs requires the careful adjustment of a variety of ingredients that can enhance or limit the desired texture and visual appearance, as well as the overall properties of food. The production of fish and meat analogs comprises careful adjustment of water, flavor, fat, binding agents, proteins, vitamins, minerals, and antioxidants with 50–80% water, which also serves as a plasticizer while processing meat substitutes and gives the finished product the appropriate juiciness [43]. Technologies used in the formation of feed are regarded as the major challenge. Processing techniques are classified into two categories: bottom-up and top-down structuring techniques. In the bottom-up approach, the end product is created by assembling individual fibers, whereas the top-down approach involves the development of fibrous structures by blending biopolymers with an external force [43].

Plant-based meat substitutes are made from a variety of ingredients, primarily from oilseeds like cottonseed and rapeseed, legumes like mung beans, common beans, and lentils, and cereals like barley, wheat, corn, oats, and rye. Legumes are a significant source of protein rich in dietary fiber, vitamins, and minerals with high antioxidant properties [44]. They are a vital part of the diet known for their effect on inhibiting diseases.

Different types of plant-based proteins have been discussed below.

#### 5.1.1. Legume-Based

##### Soy Protein

Soy protein isolate (SPI), which contains both essential and non-essential amino acids, is a significant source of protein in the human diet [45]. Being a high-quality vegetable protein, it is successfully used in 3D printing because of its self-supporting ability, water absorption, emulsification, and gelling properties [28]. The soybean is primarily used to create textured vegetable protein and gives a fibrous chewiness, hardness, and mouthfeel to the meat analog [46]. Chen et al. [47] reported that textured-soy protein (TSP) with xanthan gum showed the best printing characteristics of steak-like foods (Table 2). Also, a study showed that the printability of food inks can be improved by adding plant-based hydrocolloids, which are generally used to improve gelatinization. These additives are widely used in 3-D printing to improve the printing performance of natural food gels, which is essential for enhancing the fluidity, deposition, and lubricity of the printing material [48]. For instance, the addition of xanthan gum in soy protein isolate resulted in better rheological and textural properties. However, a high concentration of XG (0.5% *w*/*w*) resulted in poor flexibility [28]. Also, the addition of salts (NaCl, KCl, CaCl_2_, CaSO_4_) alters the properties of gel, resulting in protein aggregation and gelation. The acquired results revealed that the xanthan gum and NaCl concentration of 0.5 g/30 g and 1 g/100 mL exhibited maximum gel strength and print shape, respectively.

##### Pea Protein

**Figure 13:**
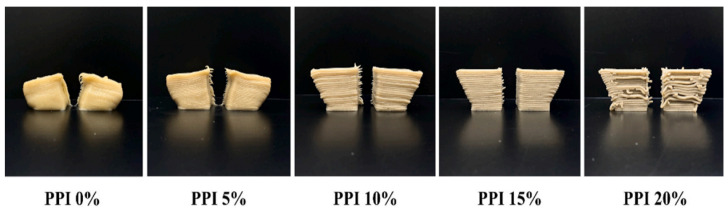
Three-dimensional printed PPI-banana pastes with different PPI concentrations (0, 5, 10, 15, 20% (*w*/*w*)) [50].

Pea protein is a hypoallergenic protein source (i.e., with low allergenicity) that is safe for consumption by people with food allergies [49]. Researchers are now focusing on development using pea protein as being a good source of fiber, starch, vitamins, minerals, and phytochemicals; however, its gelling capacity is lower than soy protein, thereby requiring the use of various additives such as hydrocolloids and salts. PPI also has a low water holding capacity and low solubility. The study carried out by Kim et al. [50] investigated the effect of different concentrations of pea protein isolate on the properties of banana-PPI paste ink. The findings of the study revealed that the incorporation of pea protein increased the protein–banana entanglement, resulting in an increase in the storage moduli (G′) and loss moduli (G″), thus improving its printability. According to the findings, banana pastes with a 15% PPI concentration could be successfully printed with a well-matched geometry and could maintain their shape after printing (Figure 13). However, a 20% PPI-induced protein aggregation in the matrix caused the 3D-printed line to break.

Another study determined the optimal alginate and pea protein ratios suitable for printing food with acceptable rheological and textural characteristics [37]. The addition of an appropriate concentration of pea protein can enhance the stability of the structure.

##### Faba and Mung Bean Protein

Faba bean proteins are known for their good emulsifying and foaming properties, but lesser than soy protein isolates [51]. However, altering the production and processing processes can improve the functionality of faba bean protein.

Mung bean proteins are becoming more and more common as a component of meat substitutes. A plant is known for both its nutritional worth and practical qualities. It has a high protein level (25–28%) and a low fat content (1–2%). A research group at the National University of Singapore produced vegan seafood using microalgae protein and mung bean protein. The team recreated the flaky, chewy, and fatty textures that seafood enthusiasts crave. A study reported optimum processing conditions to produce texturized mung bean protein using response surface methodology. This study showed great potential in mung bean protein as an alternative to meat, acting as a healthier and greener option compared to animal proteins Table 3 [52].

**Table 3:** Plant proteins and their applications in 3D food printing.

Category	Other Materials	Experimental Conditions	Results	References
Soy protein	Textured-soy protein (TSP), drawing soy protein (DSP,) xanthan gum, Konica gum, sodium alginate, guar gum, sodium carboxymethyl, cellulose	Refrigeration: 4 °C; printing nozzle temperature: 25 °C.	TSP with xanthan gum showed best printing characteristics.	[47]
	L-cysteine, Transglutaminase	pH: 7, heating: 90 °C; mixing: 1500 rpm (1 min) and 300 rpm (2 min).	SPI heated for 25 min with l-cysteine had best printability and stability.	[28]
	K-carrageenan, vanilla powder	Heating: 70 °C; microwave: 50, 80, and 110 W	SPI gel made with 3% carrageenan had the optimal viscosity for 3D printing.	[53]
	Guar gum, xanthan gum, soybean oil, NaCl powder	Homogenization: 800 rpm, 5 min; heating: 70 °C, 60 min.	SPI gel with xanthan showed better rheological properties but a high concentration of XG (0.5% *w*/*w*) resulted in poor flexibility.	[47]
	Strawberry powder	Microwave: 30, 50 and 70 W	Salt pretreatment improved the printability and shape stability of ink systems. Maximum shape accuracy—70 W.	[30]
Pea protein	Alginate, calcium chloride, sodium phosphate	Temperature: 45 °C	Alginate solution (80%) and pea protein solution (20%) were most suitable for 3D printing.	[37]
	Microwave vacuum-dried banana powder, ascorbic acid	Blending: 1 min; sifting: 300 μm; mixing: 2000 rpm, 25 °C, 6 min	Banana pastes with 15% PPI concentration retained their shape and geometry after printing.	[50]
Mung bean protein	Mung bean flour, hydrochloric acid, sodium hydroxide, Coomassie Blue R250, and bromophenol blue.	Mixing with 100 mL water; blending: pH-9, 2000 rpm, 30 °C, 1 h; centrifugation: 8586× *g*; freeze-drying: 48 h.	Optimized extrusion parameters: feed moisture: 49.33%; screw speed: 80.66 rpm; and barrel temperature: 144.57 °C; fibrous structure, partial protein unfoldment, high retention of amino acids.	[52]

#### 5.1.2. Cereal-Based

It comprises wheat, corn, oat, and rice, which are known for their high starch content. Wheat protein, also called gluten, is the most commonly used cereal-based protein, especially in the production of meat analogs, due to its viscoelastic properties [6]. Cereals have been used extensively in extrusion-based 3D printing of pizza, cookies, and dough due to their good shear stability [54].

##### Gluten Protein

Wheat is widely consumed around the world, having starch as a primary component followed by proteins and non-protein compounds such as cellulose, hemicelluloses, polyphenols, and minerals. Due to their high nutritional and organoleptic quality, wheat-based goods, such as wheat flour (flour with the bran removed) and wheat whole meal (flour with the bran included), are essential dietary components worldwide. Gluten plays a major role in 3D printing a dough and its printability can be improved by the addition of salts such as NaCl. NaCl improves the gluten protein structure stability in the dough by promoting the hydrophobic interaction and polymerization of the gluten proteins [55].

##### Oat Protein

Oat protein is known for its good amino acid concentration and has a better nutritional value of 15–20% as compared to other cereal proteins due to its high lysine content. Oat protein has a stable network even at a high denaturation temperature of 110 °C, and when mixed with soy protein, it can improve the strength of the gels [56]. For instance, 35% oat protein when mixed with 45% fava bean protein isolate printed food of the highest stability [1]. Also, a study reported that oat protein when combined with pea protein produces a good sensory effect [57].

##### Rice Protein

Rice, a known low allergenicity raw material and, in particular, promoted as a soy substitute, is a very promising raw material for producing meat analogues. In current studies, rice flour is utilized in meat products to replace fat and benefit from its ability to bind water. A study conducted by Qiu et al. indicated that adding rice protein in soy protein–wheat gluten protein pastes can significantly improve their 3D-printing properties by reducing viscosity and shear modulus.

### 5.2. Role of Additives

In 3D food printing, additives are frequently utilized to improve the printing performance of natural food gels, which is essential for enhancing the fluidity, deposition, and lubricity of printing materials [48]. Various additives like hydrocolloids (xanthan gum, guar gum) were mentioned in the previous sections. These are the most commonly used ones for the 3d printing of plant-based proteins. There are two main functions of additives—improving the stability of final 3D printed products and improving performance in other areas like health and nutrition and sustainability using alternative food sources like meat analogs. For instance, compared to traditional sources of food such as meat (beef) or fish, protein-based meat analogues that mimic traditional meat not only provide high-quality protein but also improve sustainability (reducing the need to rear animals, smaller land requirements, and less greenhouse gas emission). In this regard, the Netherlands Organization for Applied Scientific Research introduced a food that was designed for elderly people to solve their swallowing and chewing problems [58]. Patients with dysphagia have varying texture tolerances as described by the International Dysphagia Diet Standardization Initiative (IDDSI). So, in that study, hydrocolloids were added for ink optimization and alteration of texture in 3D-printed dysphagia foods [58]. Additives currently used in 3D food printing are shown in Table 4.

## 6. Post-Printing Treatments

Post-processing refers to the steps carried out after the actual printing of the food item to enhance the final product’s quality, appearance, and taste. Typically, food inks suitable for printing are either pre-processed to ensure the desired taste upon printing or pre-processed, necessitating post-treatments after deposition to guarantee edibility [61].

**Figure 14:**
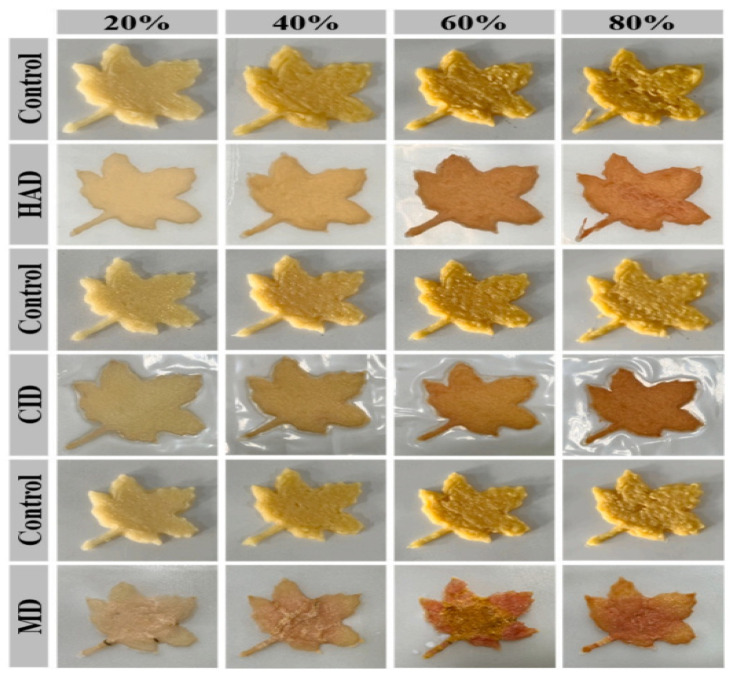
Effect of different drying methods on 3D printed C-WPI-NPs [63].

Only a small fraction of 3D-printed products do not require post-processing treatments, while most of the 3D-printed food products need post-processing, including baking, steaming, and frying, which can induce favorable alterations in the texture—an essential sensory characteristic influencing product quality and attractiveness [62]. Drying is a frequently employed post-processing approach in the field of food printing [62]. At present, various drying techniques such as freeze drying, oven drying, vacuum microwave drying, and other recently innovated methods are employed to manipulate the characteristics of 3D-printed foods [62]. Various researchers have studied the influence of different drying methods on the shape stability of 3D food products. A study reported the effect of oven drying and freeze drying on protein–cellulose based ink with different dry matter. Experimental findings indicated that the freeze-drying process of printing characterized by a low dry matter content (35%) results in a stable structure. One potential explanation for this observation is that, with an initial low dry matter content of 35%, the water content is elevated, leading to increased structural strength [1]. Another study investigated the effect of microwave drying (MD), catalytic infrared drying (CID), and hot air drying (HAD) on the color of curcumin–whey protein isolate nanoparticle (C-WPI-NP) printed samples. It was reported that CID showed a consistent and obvious red color shift, with a 92.35% retention rate in the size of the dried product [63] as shown in Figure 14.

**Figure 15:**
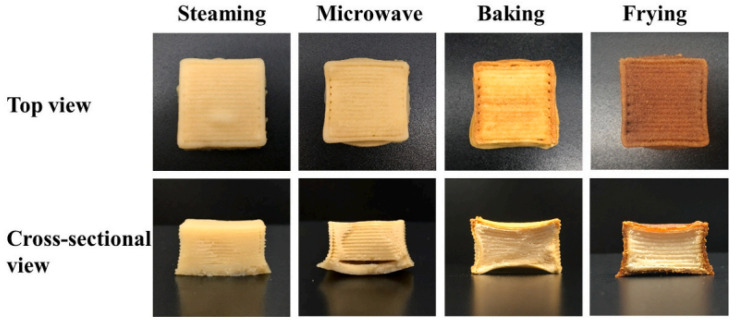
Effect of TG on different post-treatment methods of MBPI-MC meat analogues [60].

Also, in order to facilitate the widespread adoption and approval of 3D printed foods among consumers, it is essential for 3D printing technology to integrate seamlessly with conventional food processing methods such as baking, steaming, frying, and other cooking techniques. Nevertheless, a significant challenge in achieving this lies in preserving the structural stability of 3D-printed foods throughout the cooking process, which can be improved by the use of additives [64]. A study evaluated the effect of transglutaminase (TG) on the cooking loss and shrinkage of mung bean protein isolate–methylcellulose complexes (MBPI-MC). It was concluded that when comparing different cooking methods, the cooking loss and shrinkage of TG meat analogues were lower after steaming than after baking, frying, and microwaving (Figure 15) [60]. This may have been due to the high water-retention ability of the meat analogue during steaming and the formation of soluble protein aggregates [60].

## 7. Challenges and Future Perspectives

Realizing nutrition’s comprehensiveness and customization is the primary goal of 3D-printed food. These duties enable us to ensure strict product quality and accurate nutrition control to cater to the needs of people like athletes, sick, elderly, children, and pregnant women who require high-quality and readily digestible protein. The researchers should pay attention to the quality and concentration of the materials used to make 3D printing inks as they directly affect the health of humans.

The current development trend is towards developing foods for vegetarians. For that, it is important to note that various sources of plant protein such as pea, soy, and oat can be mixed together in an optimal quantity so as to be used as a potential substitute to meat protein. Animal protein does not have the same health benefits as plant protein, which in return has a longer shelf life and has plenty of nutrients, fiber, and antioxidants. Additionally, plant proteins meet the dietary requirements of vegetarians and have the potential to be used as a substitute to produce meat analogues. For instance, soy protein due to its self-supporting structure and gel-forming properties is termed as an essential plant protein to produce meat products.

Although plant protein materials show promise for 3D-printing applications, the following points need to be better understood for their use in this application.

- Printing precision and shape stability are the biggest challenges to overcome. The development of future 3D-printing inks still depends on the concentration, type, and the environmental and operating conditions which need to be controlled in accordance with the rheological properties of the food. A superior finished product is made by controlling printing parameters such as pH, temperature, speed of nozzle, nozzle diameter, and the material quality and quantity. The printability and self-supporting property of the ink is improved by incorporating various additives to the ink such as hydrocolloids, carbohydrates, lipid additives, phenolic compounds, enzymes, starches, and hydrogels. Lately, there has been a demonstration of cellulose’s potential to enhance the characteristics of emulsions based on proteins. Cellulose materials are attracting attention due to their status as the main constituent in plants. Cellulose, as a sustainable and inexhaustible polymeric raw material, has the capacity to fulfill the growing need for eco-friendly products [65]. Also, it might be effective to combine 3D food printing with other cutting-edge technology. For instance, microwave and ultrasonic technologies are applied during pre- or post-processing to enhance the printing accuracy and shape stability [30].
- Preserving the textural and sensory attributes of the printed food. Sensory attributes such as mouthfeel are influenced by product texture and its ability to bind water. The sensory and textural characteristics of food are impacted by the presence of fats. However, the prolonged excessive intake of saturated fats heightens the susceptibility to numerous chronic conditions, including obesity, cardiovascular disease, and metabolic syndrome. In recent times, nutritional awareness has grown and there is an increased focus on low-fat products. Emulsions are the potential fat replacers, and incorporating cellulose into protein emulsion-based fat replacers enhances the nutritional, textural, and sensory attributes. This improvement is attributed to cellulose’s ability to effectively retain water, stabilize interfaces/networks, and thickening effects in addition to its nutritional value as dietary fiber [66].
- Meat products are characterized by a red or pink color that is obviously hard to obtain without the application of colorants. Unfortunately, the issue still exists since many consumers who choose vegetarian goods also avoid additives, which makes the matter more technologically challenging. However, the growing use of 4D printing has encouraged a more thorough investigation into product appearance, which includes color and shape.
- Production efficiency. The size and speed of 3D food printing prevent its usage in industrial-scale food production. Although the printing speed or nozzle diameter can be increased, doing so frequently leads to a loss of printing resolution. Researchers have suggested speeding up printing by using adaptive algorithms, which might change the printing settings to balance the printing quality and time [48]. Using multi-nozzle printers to print multiple 3D objects at once is another possible strategy. Future studies should look into the incorporation of phenolic compounds such as flavonoids, as they are closely related to the sensory and nutritional quality of the food. Future research must examine these issues and opportunities for plant-protein-based inks.
- Consumer acceptance: Acceptability and pleasantness of 3D-printed food is one of the major challenges. A study conducted by Lupton et al. [67] reported the concerns of many participants that the food created using a printer might be inedible, unsafe, or nutritionally deficient. Additionally, the term ‘printer,’ typically linked with non-food industries, appeared to negatively influence participants’ willingness to accept such technology. Ross et al. [68] conducted a study on Irish people and reported that the attitudes of consumers towards the use of 3D food printing technologies might differ depending on the consumer’s country of residence. A study revealed that consumer acceptance to 3D-printed food depends on (1) the initial information provided, i.e., the first impression consumers receive, and that (2) well-designed communication has the potential to positively shape consumers’ attitudes toward 3D-printed food [69].

## 8. Conclusions

This review article entails the virtually new concept of personalized nutrition called 3D food printing. This is a new and innovative field having the potential to customize the design, nutrition, and composition of food products. A focus was placed on plant-protein-based inks given their wider usage in research, as compared to animal proteins. One of the most diverse applications of plant proteins is to produce meat analogs. Consumers are becoming vegetarians or seeking out goods that are not made from animal products at an increasing rate today. The majority of meat substitutes contain soy and wheat-derived proteins, such as gluten. Although plant-based beef burgers and sausages have been used successfully, most of these recipes use minced meat instead of whole-cut meat fillets, which lack their distinctive appearance. This might be as a result of the extrusion processing methods used for the current plant-based meat substitutes that create a product having a consistent appearance. Although plant proteins are frequently acknowledged as a sustainable substitute for animal proteins, care must also be taken to minimize their harmful effects on the environment during their extraction. Additionally, it was also discovered that hydrocolloids and other additives had significant roles in the production of plant-protein-based printable gels. As we explore new sources of protein to fulfill the needs of a growing population, the demand for plant-based protein will undoubtedly rise in the coming years. Despite the breakthroughs in 3D food printing technology, the issues of providing comprehensive nutrition and personalization, rational protein extraction techniques, improving printing precision and accuracy, and paying attention to the appearance and texture of the finished product still exist.

## Figures and Tables

**Table 1:** Effect of printing speed and extruding rate on the printability of WPI [13].

Ingredient Ratio (w:w:w:w)					Oil Content (%, *w*/*w*)	Manufacturer-Defined Printing Speed (Actual Printing Speed, mm/s)	Manufacturer-Defined Extruding Rate (Actual Extruding Rate, mm^3^/s	Printed Shape	Printing Quality Score
No.	CS	W	CO	WPI					
A	20	25	25	25	26.3	100 (21.1)	100 (20.0)	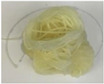	1
B	22	25	25	25	25.8	100 (21.1)	100 (20.0)	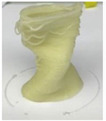	3
C	25	25	25	25	25.0	100 (21.1)	120 (26.8)	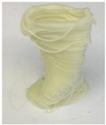	3
D	25	25	25	22	25.8	100 (21.1)	110 (23.3)	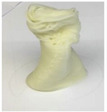	2
E	25	25	25	20	26.3	100 (21.1)	100 (20.0)	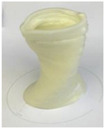	4
F	25	28	25	25	24.3	100 (21.1)	100 (20.0)	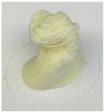	2

(CS: corn starch; W: water; CO: canola oil; WPI: whey protein isolate).

**Table 2:** Printing results of textured-soy protein (TSP) and drawing-soy protein (DSP) using different hydrocolloids [47].

Protein	Control	Guar Gum	Sodium Alginate	Hydroxyethyl Cellulose	Xanthan Gum	Sodium Carboxymethyl Cellulose	Konjac Gum
Textured Soybean Protein	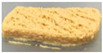	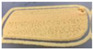	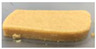	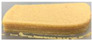	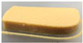	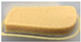	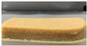
Drawing Soy Protein	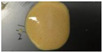	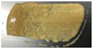	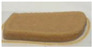	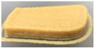	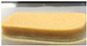	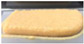	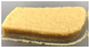

**Table 4:** Recent applications of additives in 3D printing of plant-based proteins and main changes in printing characteristics.

Types	Additives	Materials	Finding		References
Hydrocolloids	Alginate	Pea protein powder (PP), calcium chloride	Increased gel strength.	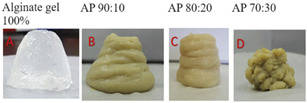	[37]
	Agar	Soy protein acid hydrolysate (SPAH)	Improved mechanical strength and increased self-supporting capacity of 3D printed structures.	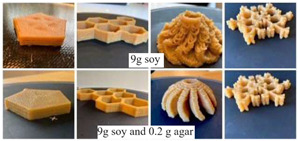	[33]
	Kappa-carrageenan	Soy protein isolate (SPI), vanilla powder (for flavor)	3D printed structures with smooth surfaces and denser gel network structures.	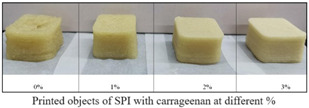	[53]
	Xanthan gum (XG)	Pea protein isolate (PPI)	A small amount of XG improved mechanical strength and chewing and swallowing easiness.	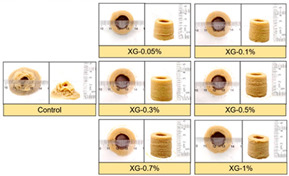	[59]
Others	Transglutaminase (T_Gase_) powder	Mung bean protein isolate (MBPI), methylcellulose (MC)	Smooth printed surface, improved mechanical strength, increased hardness. Optimal TG: 4 U/g of MBPI.	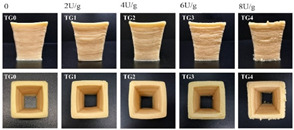	[60]

## Data Availability

The data that support the findings of this study are available from the corresponding author upon reasonable request.
